# Fragment docking supported by NMR shift perturbations

**DOI:** 10.1186/1758-2946-6-S1-P18

**Published:** 2014-03-11

**Authors:** Tim ten Brink, Clementine Aguirre, Isabelle Krimm

**Affiliations:** 1 Institute des Sciences Analytiques – CNRS UMR5280 , Universite Claude Bernard - Lyon 1, Villeurbanne, F-69622, France

## 

Fragment-based approaches have become popular tool in drug design due to their ability to screen large portions of chemical space with comparatively small libraries. However fragments can exhibit unspecific binding and even if they bind to a specific binding site in some cases more than one binding mode is observed [1]. For computational approaches like molecular docking fragments pose also new challenges. Score differences between different binding modes generated by docking are often small, making the identification of the correct, natural binding mode difficult.

The sensitivity of a nuclei's NMR chemical shift to changes in its chemical environment can be used to measure chemical shift perturbations (CSP) of protein atoms upon ligand binding. Especially ^1^H and ^15^N CSP are easily obtainable from ^15^N HSQC spectra and can be used as probe for ligand orientation but also include information about conformational changes on the protein side. CSP data has been used to orientated drug like molecules into protein binding sites [2,3] and can be included into the scoring function to improve docking [4].

Here we show how CSP can be used to quickly validate docking poses of smaller fragments by filtering them for their agreement between experimental CSP and simulated CSP for the docked poses. Additionally a more detailed analysis of the differences between the experimental and the simulated CSP profiles can be used to highlight protein regions and even single residues which undergo structural changes upon fragment binding.
